# African Queens find mates when males are rare

**DOI:** 10.1002/ece3.9956

**Published:** 2023-04-02

**Authors:** Vincent P. Rutagarama, Piera M. Ireri, Constantin Sibomana, Kennedy S. Omufwoko, Simon H. Martin, Richard H. ffrench‐Constant, Winnie Eckardt, Beth K. Kaplin, David A. S. Smith, Ian Gordon

**Affiliations:** ^1^ Department of Biology, College of Science and Technology University of Rwanda Kigali Rwanda; ^2^ International Centre for Insect Physiology and Ecology Nairobi Kenya; ^3^ Department of Ecology and Evolutionary Biology Princeton University Princeton New Jersey USA; ^4^ Institute of Ecology and Evolution, School of Biological Sciences University of Edinburgh Edinburgh UK; ^5^ Centre for Ecology and Conservation University of Exeter Penryn UK; ^6^ Dian Fossey Gorilla Fund Musanze Rwanda; ^7^ Center of Excellence in Biodiversity & Natural Resource Management University of Rwanda Butare Rwanda; ^8^ Natural History Museum, Eton College Windsor UK

**Keywords:** *Danaus chrysippus*, male killing, sex ratio, spermatophore, *Spiroplasma*

## Abstract

In butterflies and moths, male‐killing endosymbionts are transmitted from infected females via their eggs, and the male progeny then perish. This means that successful transmission of the parasite relies on the successful mating of the host. Paradoxically, at the population level, parasite transmission also reduces the number of adult males present in the final population for infected females to mate with. Here we investigate if successful female mating when males are rare is indeed a likely rate‐limiting step in the transmission of male‐killing *Spiroplasma* in the African Monarch, *Danaus chrysippus*. In Lepidoptera, successful pairings are hallmarked by the transfer of a sperm‐containing spermatophore from the male to the female during copulation. Conveniently, this spermatophore remains detectable within the female upon dissection, and thus, spermatophore counts can be used to assess the frequency of successful mating in the field. We used such spermatophore counts to examine if altered sex ratios in the *D. chrysippus* do indeed affect female mating success. We examined two different field sites in East Africa where males were often rare. Surprisingly, mated females carried an average of 1.5 spermatophores each, regardless of male frequency, and importantly, only 10–20% remained unmated. This suggests that infected females will still be able to mate in the face of either *Spiroplasma*‐mediated male killing and/or fluctuations in adult sex ratio over the wet–dry season cycle. These observations may begin to explain how the male‐killing mollicute can still be successfully transmitted in a population where males are rare.

## Graphical abstract

African Queens or Monarchs can still find males even in the face of extensive male‐killing. This finding has unexpected implications for the fitness and maintenance of the male‐killing endosymbiont *Spiroplasma*.

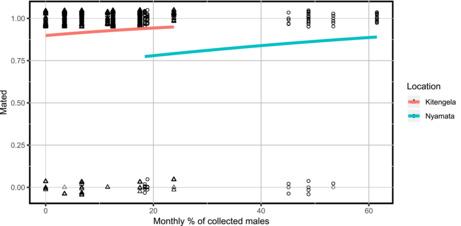

## 1 INTRODUCTION

The evolutionary consequences of highly distorted sex ratios in insects, for example, as mediated by selfish endosymbionts, are a subject of extensive ongoing debate (Charlat et al., 2003; Duplouy & Hornett, 2018; Engelstadter & Hurst, 2007). One strategy suggested to reduce the risks and costs of such selfish endosymbionts is the evolution of multiple matings in females or polyandry (Wedell, 2013). In turn, polyandry itself can affect gene flow, effective population size, and therefore, host population viability (Holman & Kokko, 2013; Wedell, 2013). These factors result in a dynamic interaction between the effects of selfish endosymbionts and polyandry, generating both sexual selection and conflict within the infected host (Wedell, 2013). In field populations, the simplest consequence of a highly female‐biased sex ratio is that females may remain unmated, as virgins, potentially leading to population decline. This situation is complicated in low‐density insect populations where mating failure can be a typical cause of Allee effects in the absence of any male killing (Rhainds & Fagan, 2010; Robinet et al., 2008). Model field‐based systems in which we can begin to disentangle the effects of selfish endosymbionts, polyandry, and Allee effects are rare, and one long‐term aim of the current study is to establish the African Monarch, *Danaus chrysippus*, as a system in which we can begin to disentangle these effects.

To examine the effects of male killing, and/or sexual asynchrony caused by migration, we need to determine the operational sex ratio (OSR) in the field. The OSR is defined as the proportion of males in the adult population, and it influences a wide range of ecological factors such as mating behavior (Ancona et al., 2017; Jiggins et al., 2000). OSRs fluctuate over time both for intrinsic reasons such as male‐kiling and for extrinsic factors such as sex‐specific migration, as well as potential differences in the behavior of males and females (Ancona et al., 2017). Despite the difficulty in estimating OSRs in the field, in butterflies we can accurately determine the frequency of successful matings via the detection of spermatophores in mated females. During mating, male butterflies transfer a spermatophore, containing both sperm and accessory factors, to the receptive female (Cardoso & Gilbert, 2007; Sanchez & Cordero, 2014). As the chitinous neck of the spermatophore remains in place after copulation, the presence or absence of one or more spermatophores in the bursa copulatrix of females can be used to determine the frequency of successful mating (or at least successful spermatophore transfer) in the field (Burns, 1968). In Lepidoptera, the delivery of sperm from the spermatheca is further complicated by the presence of both functional sperm (eupyrene) and parasperm (apyrene sperm) whose role in Monarch butterflies is unclear. Parasperm could play a role in facilitating the success of the eusperm, provisioning the female or zygote and/or mediating postcopulatory sexual selection (Wedell et al., 2009; Whittington et al., 2017). However, the ratio of functional sperm to parasperm is not known in our study species, and we therefore simply use the presence of one or more spermatophores in field‐collected females to document the observed levels of successful mating (the frequency of females carrying one or more spermatophores) and the apparent frequency of multiple mating (the number of spermatophores found in females that have re‐mated, where each spermatophore represents mating with one male).

Previous observations in butterfly populations with a high frequency of maternally inherited male‐killing endosymbionts, such as *Wolbachia* or *Spiroplasma*, show that a scarity of males can indeed increase the percentage of virgins found in a field population. For instance, 94% of female *Acrea encedon*, infected with a male‐killing *Wolbachia*, remained as virgins in a Ugandan population (Jiggins et al., 2000). Similarly, 50% of female *Hypolimnas bolina* in Samoa, infected with a different male‐killing *Wolbachia*, remained unmated (Dyson & Hurst, 2004). Male killing can, in theory, benefit the parasitic endosymbiont through increased resource availability to infected female offspring (in the absence of their brothers), thereby increasing the transmission to future generations (Hurst et al., 1993). However, a failure of infected females to find mates, when sex ratios are strongly female‐biased, could also impose a cost of the male‐killing phenotype on the endosymbiont itself. Despite this potential cost to the male killer, infected Samoan populations of *H. bolina* have persisted for over 100 years despite a highly female‐biased sex ratio of 100:1 (Dyson & Hurst, 2004). In fact, this apparently stable situation has only recently been disrupted by the emergence of host resistance to male killing on the island (Hornett et al., 2014; Reynolds et al., 2019). The suggested presence of this fitness cost to the male‐killing microbe, in the form of unmated females that fail to transmit the male killer, therefore makes untested assumptions about the relative efficiency of males and females in finding mates in the face of female‐biased sex ratios.

*Danaus chrysippus* is found throughout Africa as a series of different color morphs or subspecies. These color morphs converge on East Africa where admixture polymorphism, driven by the winds surrounding the Inter‐Tropical Convergence Zone (ITCZ), creates populations with all color morphs present. This zone of admixture has been termed a contact zone for the different subspecies, and within that contact zone, some individuals are infected with a male‐killling mollicute called *Spiroplasma* (Smith et al., 2019). Previous work has shown that *Spiroplasma* infection is linked to the occurrence of a *neo‐W* sex chromosome and associated changes in color pattern, and that both the endosymbiont and the *neo‐W* are strictly maternally inherited (Martin et al., 2020; Smith et al., 2019). There therefore appears to have been a single infection of the *neo‐W* carrying lineage (Martin et al., 2020) and subsequent spread of this *Spiroplasma‐neo‐W* complex across a region centered on southern Kenya in East Africa (Smith et al., 2019). This zone of infection overlaps with the broader contact zone where the different color morphs of *D. chrysippus* meet and inter‐breed (Liu et al., 2022; Smith et al., 2019). In some parts of this contact zone, sex ratios are highly female‐biased (Smith et al., 2019), raising the possibility that the scarcity of males may result in a failure of females to mate. In other areas, both infection rates and sex ratios vary between the wet and dry seasons (Hassan et al., 2013; Herren et al., 2007), possibly associated with seasonal migration of the different color morphs (Smith et al., 1997).

Here, we therefore sampled two East African populations carrying differing frequencies of the male‐killing mollicute *Spiroplasma* (Jiggins et al., 2000) and showing dramatic seasonal variation of OSR, in order to test if a lower proportion of males leads to a lower frequency of mated females associated with a failure of females to find mates. At the first site, Kitengala in Kenya, male killing is prevalent (Smith et al., 2019), whereas at the second site, Nyamata in Rwanda, *Spiroplasma* infection rates currently still appear low (Ndatiman et al., 2022). Strikingly, we find that mating rates are unaffected by OSR variation at both sites, and that females still mate (receive one or more spermatophores) when males are rare. While the relative importance of male killing and sex‐specific dispersal in these two populations remains unresolved, the fact that infected females can still find male mates suggests that there may currently be little localized fitness cost of male killing to the *Spiroplasma* endosymbiont itself. Together, these findings help explain how the male killer can persist in highly female‐biased populations.

## 2 MATERIALS AND METHODS

### 2.1 Study sites and sampling

Butterflies were collected at two different field sites in East Africa, both within the *D. chrysippus* contact zone where the different color morphs fly together. The first, Kitengala, near Nairobi in Kenya, is known to harbor high frequencies of the male‐killing *Spiroplasma* and to consistently display female‐biased sex ratios with a mean of 74.5% females (Smith et al., 2019). The second site, Nyamata in Rwanda, is highly polymorphic for butterfly color pattern while infection rates still appear low (Ndatiman et al., 2022). Sampling at Nyamata was carried out throughout 2021 only (*N* = 787), while at Kitengela, butterflies (*N* = 304) were collected between May 2013 and July 2014. Adult males and females were collected using a butterfly net and a random subset of females (all females if <10 collected per month or 10 randomly selected females if more than 10 were collected per month) was selected for dissection (Nyamata dissected *N* = 102 and Kitengela *N* = 250). A successful mating was defined as the presence of one or more spermatophores in a single dissected female. Mating rates were defined as the number of spermatophores found in mated females (where each spermatophore is inferred to come from a mating with a different male).

### 2.2 Statistical analyses

**FIGURE 1:**
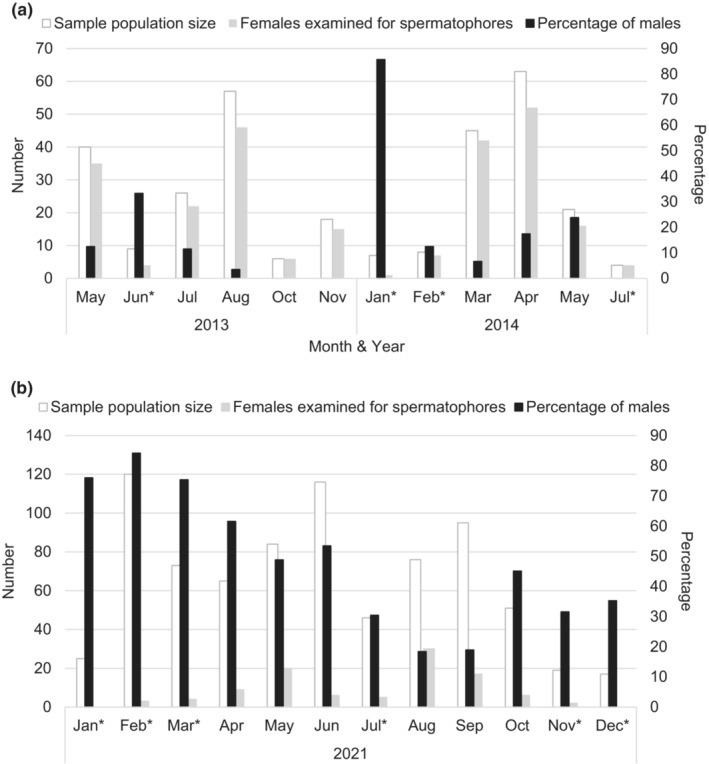
Number of butterflies collected, number of females examined for spermatophores, and percentage of males in the sample population by month and study site: (a) Kitengela, Kenya, and (b) Nyamata, Rwanda; *Indicating months excluded from data analysis due to small sample size.

Adult sex ratio, or OSR, was calculated as the percentage of males in the sample population for each month and site (Figure 1a,b). To examine the possible effect of OSR on the frequency of successful matings, we ran two different generalized linear models (GLMs), using the ‘glm2’ function in the R Statistical Software (v4 1.2; R Core Team, 2021). The first GLM, used a binomial error structure and tested whether the mating success of a female (yes/no, as indicated by the presence/absence of any spermatophores) can be explained by the monthly percentage of collected males in a population. The second GLM used a Poisson error structure and examined whether the number of spermatophores in each female can be explained by the monthly percentage of males in a population. Both models also included study site as a predictor variable. We also tested the interaction terms between location and monthly percentage of collected males, which were not significant in either model, and were therefore excluded from the final model itself. Months in which <10 butterflies were collected at either site, or less than six females dissected for spermatophores, were excluded from the analysis (Figure 1a,b for numbers of butterflies used in the analysis). We checked the Poisson model for under or over‐dispersion, but no issues were detected.

## 3 RESULTS

Monthly adult sex ratio, presented as the percentage of males collected (Figure 1a,b), varied greatly at each location (full dataset Kitengela: mean = 17.3%, range: 0–85.7%; full dataset Nyamata: mean = 48.3%, range: 18.9–84.2%). Spermatophore counts from the full dataset showed that Rwandan females mated on average 1.37 ± 1.08 times (range: 0–5, *N* = 102), whereas Kenyan females mated on average 1.72 ± 0.93 times (range: 0–6, *N* = 250).

**FIGURE 2:**
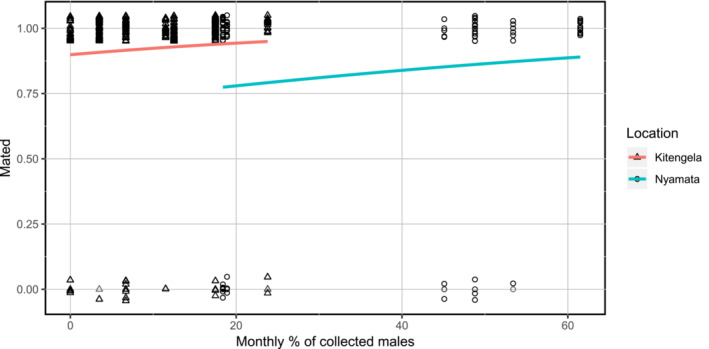
Jitter plot showing the distribution of mated females (jittered around 1) and unmated females (jittered around 0) by monthly percentage of collected males and study location. Lines indicate fitted logistic regression curve by location.

**FIGURE 3:**
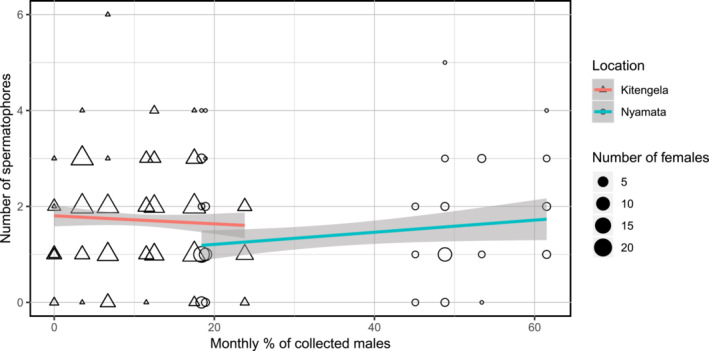
Relationship between number of spermatophores and the proportion of males in monthly samples at both study locations. The size of circles and triangles depicts the number of females in each monthly sample. Lines indicate fitted regression curve by location, and gray shading indicates 95% confidence interval.

GLMs based on the reduced dataset (containing only months with sufficient sample sizes), including the interaction term between monthly percentage of males collected and location, indicated that location does not significantly affect model predictions for successful female mating (est = 0.014 ± 0.009, *z* = 1.525, *p* = .127) or indeed how often females mated (est = −0.014 ± 0.041, *z* = −0.338, *p* = .735). After rerunning both GLMs without the interaction term, we found that the percentage of males in a population is not a significant predictor of whether a female is found to be unmated (est = 0.022 ± 0.016, *z* = 1.370, *p* = .171, Figure 2), nor of the number of spermatophores detected in each female (a useful proxy for mating frequency) (est = 0.004 ± 0.004, *z* = 0.998, *p* = .318, Figure 3), despite the considerable monthly variation in apparent male presence (Kitengela: 0–23.8%, mean = 9.4%, *N* = 8; Nyamata: 18.4–61.5%, mean = 42.5%, *N* = 6). Cross‐site comparison shows that there were significantly fewer unmated females in the Rwandan than the Kenyan sample (est = −1.460 ± 0.494, *z* = −2.955, *p* = .003), whereas overall mating frequency was found to be significantly lower in Rwanda than Kenya (est = −0.138 ± 0.148, *z* = −2.148, *p* = .032).

## 4 DISCUSSION

We wanted to test if the apparent scarcity of males in East African populations of the African Monarch can reduce mating success in female butterflies. Here, despite sampling limitations, we have shown that females at two different sites can still find mates even when males are rare. Here, we define rare as a low proportion of captured males relative to captured females. While we are currently unable to precisely dissect the relative contribuitons of male killing and seasonal migration in Operational Sex Ratio (OSR) fluctuations at esch site, our findings clearly show that mating success is not likely to be a limiting factor in the spread of the male‐killing mollicute *Spiroplasma*. This may help explain why the current *neoW‐Spiroplasma* complex appears to be spreading in East Africa, despite the fact that males are rare in some infected populations and at specific times in the wet–dry season cycle.

In the interpretation of our results, we hypothesize that OSRs differ in both time and space due to both intrinsic (e.g., male‐killing) and extrinsic (e.g., sex‐specific migration) factors and also due to likely behavioral differences in the apparency of males and females. Thus, while we cannot validate the precise accuracy of our OSR estimates, and while the periods sampled differ at each site, we have convincingly shown that males are rare at both sites at certain times of the wet–dry season cycle. Strikingly, despite the apparent scarcity of males at some times of year, the number of spermatophores per female (mean of ~1.5 per female) remains remarkably constant. There is therefore no clear relationship between female mating success and sex ratio at either site sampled. Without precise data on the relative roles of male killing (PCR determined rates of *Spiroplasma* infection) and sex‐specific migration (differential movement of males and females in and out of the study sites), it is difficult to precisely interpret the small differences in mating frequencies observed between the two sites. Thus, we cannot determine if females need to mate multiply to achieve full fecundity (Wiklund et al., 2001) or if female choice plays a role in driving the different mating rates observed. However, we do note that the higher mating rate in Kenyan butterflies is consistent with theory that suggests that polyandry may evolve to overcome issues with sperm limitation (Wedell, 2013). In turn, the Rwandan population was often male‐biased, with mating rates still remaining low, and this is not consistent with the idea that male harassment drives mating rates (Andersson et al., 2000). These complications aside, however, our finding that only 11% of females remain unmated in the face of extensive male killing by *Spiroplasma* at Kitengela, Kenya, contrasts markedly with the 50–90% of unmated females found in other male‐killer‐infected species (Dyson & Hurst, 2004; Jiggins et al., 2000). Taken together, our results therefore suggest that female African Queens can still find mates even when males appear rare, for whatever extrinsic (ecological) or intrinsic (male killing) reason.

To fully understand the likely effects of female mating success on male‐killer fitness, we need to take our previous findings into account. In brief, previous work has uncovered the recent emergence (~2200 years ago) of a single *Spiroplasma* infection, linked to a single origin of a new female‐limited sex chromosome, here termed simply the *neo‐W* (Martin et al., 2020; Smith et al., 2019). The *neo‐W* and *Spiroplasma* are maternally co‐inherited, forming a *neo‐W‐Spiroplasma* ‘complex’ that shows a surprisingly high frequency in the eastern parts of the hybrid zone between the different *D. chrysippus* subspecies (Martin et al., 2020). We expect that the highly female‐biased sex ratios generated by the *neo‐W‐Spiroplasma* complex should result in an increase in the proportion of unmated female butterflies, as discussed above. As *Spiroplasma* is transmitted from mated females to their infected eggs, the presence of excess virgin females in a population should theoretically lead to a decline in the infected female population and potentially also limit the rate at which male killing can spread. This prediction, however, is based on the assumption that some females will not find mates when sex ratios are highly female‐biased. Given that only 10–20% of females at our two study sites remain unmated, regardless of the proportion of males, there may in fact be no detectable cost of male killing to the *Spiroplasma* endosymbiont itself. The efficiency with which females appear to be able to find and/or attract males may therefore help explain why male killing has spread to such high frequencies in some parts of East Africa. It also suggests that theory developed around local extinction driven by male killing (Hassan et al., 2013) may not actually apply to the African Queen‐*Spiroplasma* system. However, our findings still do not explain why the *neo‐W‐Spiroplasma* complex is apparently confined to a region within, but not perfectly overlapping with, the contact zone between the *D. chrysippus* subspecies. This suggests that other ecological or genetic forces, such as elevation, migration, or host resistance, might be at work to contain the spread of the male killer.

More precise determination of the relative roles of male killing and migration at each site should in turn facilitate more detailed assessments of the roles of male harassment and/or female choice in driving observed mating rates. Further, and critically, we need to directly compare spermatophore counts between infected and un‐infected females, as detected via PCR. Thus, at present, we cannot distinguish between the two alternative hypotheses that may explain our results. First, that the effect of male abundance of female mating success is simply an Allee effect driven by the difficulty of finding males when they are at low density. Second, that fluctuations in the OSR drive mating success or polyandry. Despite these alternative explanations for our results, the simple fact remains that females can still find mates even when males are rare, regardless of our current inability to differentiate between the two alternative explanatory hypotheses. In conclusion, here we have shown that butterfly spermatophore counts provide an accurate read‐out of mating success (successful transfer of one or more spermatophores) and indeed mating frequency (number of spermatophores recorded per female). Using such counts, we find that female African Queens can still find mates when males are rare. This may begin to explain why the male‐killing *Spiroplasma* can still spread across East Africa when paradoxically it is also reducing the number of males that infected females can mate with. Further work will now seek to define the exact factors that determine fluctuations in the OSR at the two different field sites and, given the apparent lack of fitness cost to the parasite, to explain why male killing is currently confined to only a subsection of the East African contact zone.

## AUTHOR CONTRIBUTIONS

**Vincent Rutagarama:** Investigation (equal). **Piera Ireri:** Investigation (equal); methodology (equal). **C. Sibomana:** Investigation (equal). **Kennedy Omufwoko:** Investigation (equal); methodology (equal). **Simon Martin:** Investigation (equal); methodology (equal); writing – original draft (equal); writing – review and editing (equal). **Richard ffrench‐Constant:** Writing – original draft (equal); writing – review and editing (equal). **Winnie Eckardt:** Software (equal); supervision (equal); validation (equal); writing – original draft (equal). **Beth Kaplin:** Writing – original draft (equal); writing – review and editing (equal). **David Smith:** Writing – original draft (equal); writing – review and editing (equal). **Ian Gordon:** Investigation (equal); methodology (equal); project administration (equal); supervision (equal); validation (equal); visualization (equal); writing – original draft (equal); writing – review and editing (equal).

## FUNDING INFORMATION

This study was financially supported by a University Research Fellowship to S.H.M. and a Merit Award to R.ff.‐C, both from the Royal Society of London, grants from the National Geographic Society (to I.G., D.A.S., Rff‐C and S.H.M) and the Cleveland Metroparks Zoo (to V.R.), and the continued generous financial support of Maaike Manten.

## Data Availability

Sex ratio, spermatophore numbers and R code: Figshare doi:10.6084/m9.figshare.21947729.
